# Physician Use of Stigmatizing Language in Patient Medical Records

**DOI:** 10.1001/jamanetworkopen.2021.17052

**Published:** 2021-07-14

**Authors:** Jenny Park, Somnath Saha, Brant Chee, Janiece Taylor, Mary Catherine Beach

**Affiliations:** 1 Oregon Health and Science University, Portland; 2 Division of General Internal Medicine, Johns Hopkins School of Medicine, Baltimore, Maryland; 3 Center to Improve Veteran Involvement in Care, VA Portland Health Care System, Portland, Oregon; 4 Applied Physics Laboratory, Johns Hopkins University, Baltimore, Maryland; 5 Johns Hopkins School of Nursing, Johns Hopkins University, Baltimore, Maryland; 6 Berman Institute of Bioethics, Johns Hopkins University, Baltimore, Maryland; 7 Center for Health Equity, Johns Hopkins University, Baltimore, Maryland

## Abstract

**Importance:**

Negative attitudes toward patients can adversely impact health care quality and contribute to health disparities. Stigmatizing language written in a patient’s medical record can perpetuate negative attitudes and influence decision-making of clinicians subsequently caring for that patient.

**Objective:**

To identify and describe physician language in patient health records that may reflect, or engender in others, negative and positive attitudes toward the patient.

**Design, Setting, and Participants:**

This qualitative study analyzed randomly selected encounter notes from electronic medical records in the ambulatory internal medicine setting at an urban academic medical center. The 600 encounter notes were written by 138 physicians in 2017. Data were analyzed in 2019.

**Main Outcomes and Measures:**

Common linguistic characteristics reflecting an overall positive or negative attitude toward the patient.

**Results:**

A total of 138 clinicians wrote encounter notes about 507 patients. Of these patients, 350 (69%) were identified as female, 406 (80%) were identified as Black/African American, and 76 (15%) were identified as White. Of 600 encounter notes included in this study, there were 5 major themes representing negative language and 6 themes representing positive language. The majority of negative language was not explicit and generally fell into one or more of the following categories: (1) questioning patient credibility, (2) expressing disapproval of patient reasoning or self-care, (3) stereotyping by race or social class, (4) portraying the patient as difficult, and (5) emphasizing physician authority over the patient. Positive language was more often more explicit and included (1) direct compliments, (2) expressions of approval, (3) self-disclosure of the physician’s own positive feelings toward the patient, (4) minimization of blame, (5) personalization, and (6) highlighting patient authority for their own decisions.

**Conclusions and Relevance:**

This qualitative study found that physicians express negative and positive attitudes toward patients when documenting in the medical record. Although often not explicit, this language could potentially transmit bias and affect the quality of care that patients subsequently receive. These findings suggest that increased physician awareness when writing and reading medical records is needed to prevent the perpetuation of negative bias in medical care.

## Key Points

**Question:**

What types of stigmatizing language are written by physicians about patients in their medical records?

**Findings:**

This qualitative study of 600 encounter notes from 138 physicians found 6 ways that physicians express positive feelings toward patients in medical records, including compliments, approval, and personalization. This study also found 5 ways that physicians express negative feelings toward patients, including disapproval, discrediting, and stereotyping.

**Meaning:**

These findings suggest that physicians should increase their awareness of stigmatizing language in patient records to ensure that their notes are informative and respectful.

## Teaser

This qualitative study examines both negative and positive attitudes expressed by physicians about patients in electronic medical records.

## Introduction

Patients are not treated equally in our health care system: some receive poorer quality of care than others based on their racial/ethnic identity,^1,2,3,4^ independent of social class. Others, such as older adults^5,6^ and individuals with low health literacy,^7,8^ obesity,^9,10^ and substance use disorders^8^ may also be viewed negatively by health professionals in a way that adversely impacts their health care quality. Implicit bias among clinicians is one factor that perpetuates these disparities.^3,11,12^ Implicit bias is the automatic activation of stereotypes, which may override deliberate thought and influence one’s judgment in unintentional and unrecognized ways,^1^ and may affect treatment decisions.^4^

Literature from the field of social psychology finds that attitudes can be reflected through people’s language.^13,14,15,16^ For example, a national study of 655 emergency medicine physicians found that those who used the term “sickler” were more likely to have negative attitudes toward patients with sickle cell disease^17^ and that these negative attitudes were associated with lower physician adherence to national guidelines for pain management and medication-prescribing behavior.^20^ Biased language can in turn affect the attitudes of others hearing or reading that language. Kelly et al^21,22^ found that physicians who read a vignette with the term “substance abuser,” as opposed to “having a substance use disorder,” agreed more that the person was personally culpable and should be punished, and agreed less that the person needed treatment.

Perhaps most concerning, biased language can influence the quality of care patients receive. A 2018 randomized controlled vignette study examined how language in the medical record of a hypothetical patient with sickle cell disease would influence physicians who read the note.^23^ Readers of stigmatizing (vs neutral) language had more negative attitudes toward the patient and opted to administer less analgesia, even though all clinically relevant information was the same.^23^ These studies collectively suggest that bias can be perpetuated through patient medical records and can influence subsequent clinician attitudes and decision-making.

Understanding the ways in which bias might manifest in the language used in medical records, and developing interventions to eliminate biased language, could have a large impact on the reduction of disparities for stigmatized groups. To our knowledge, no studies have provided a comprehensive description of the types of language that might influence subsequent clinicians to respond negatively or positively. In this current study, we seek to fill that gap by identifying and describing patterns of physician language in encounter notes that have potential to transmit either negative or positive attitudes toward the patient from one clinician to another.

## Methods

### Study Participants, Setting, and Data Collection

In early 2019, we abstracted all patient medical records that had been written by physicians (attendings and residents) in 2017 at an ambulatory internal medicine setting at an urban academic medical center. From this pool of 10 550 encounter notes, we randomly selected 600 for qualitative analysis of linguistic features. All extracted notes were stored on a secure virtual environment, with access limited to study team members. The data abstracted included demographic data about patients (with its associated inaccuracies such as restriction to binary gender and inconsistent data collection methods for race/ethnicity), but the electronic medical record did not contain any demographic data about the physicians, nor was there a designated field that indicated whether the clinician was a resident or attending physician. The study was approved with a waiver of informed consent by the Johns Hopkins University institutional review board. This study followed the Standards for Reporting Qualitative Research (SRQR) reporting guideline.

### Qualitative Analysis

Throughout 2019 to 2020, we performed a content analysis of the unstructured, free text section of patient medical records. Content analysis as a qualitative method “focuses on the characteristics of language as communication with attention to the content or contextual meaning of the text,” and involves “examining language intensely for the purpose of classifying large amounts of text into an efficient number of categories that represent similar meanings.”^24^ Our goal was to discern themes, or patterns, of language used by clinicians in their encounter notes in order to define categories of language that reflected negative and positive attitudes toward patients. Our research team included 2 physicians, 1 nurse-scientist, 1 premedical student, and 1 computer scientist with expertise in natural language processing. Encounter note text was delivered to the research team in a MS Excel workbook to a secure analytic virtual environment that could only be accessed by study team members.

Two authors (J.P. and M.C.B.) read the first 100 notes and documented instances of language potentially reflecting the writer’s attitudes or opinions about the patient and that might in turn shape a reader’s attitudes toward the patient. We decided to review notes in sets of 100 because relevant language (potential positive or negative valance) that tended to yield approximately 20 notes with one or more relevant sections of text (often 30 to 50 sections of text total). Using a conventional (inductive) approach to content analysis,^24^ we reviewed notes without preconceived categories and abstracted each section of text that seemed to have any emotional valence—positive or negative—into a word processing document for discussion with the team. We took note of which emotion the text seemed to convey, and what the language seemed to be implying about the patient. Negative emotions included categories such as frustration, anger, irritation, and judgment. Positive emotions included pride, admiration, personal investment, and happiness.

After this first round, the study team met to discuss the examples and themes that were emerging from language reflecting negative and positive emotions. Using these themes, 2 authors (J.P. and M.C.B.) continued reviewing additional sets of 100 notes, met periodically together and with the rest of the team to compare our assessments, discussed the common linguistic patterns and characteristics that reflected a non-neutral impression of the patient, and occasionally modified the scope of each theme. Through this process, we refined and consolidated emerging themes into categories of positive and negative language, based on the linguistic and interpretive features of the text. After 5 rounds of reviewing 100 notes (500 notes total), the entire team had reached consensus on the major themes (categories) of negative and positive language. Two authors (J.P. and M.C.B.) then reviewed one additional set of 100 notes without further themes emerging, which suggested that we had reached thematic saturation.

## Results

### Study Participants

A total of 138 clinicians (attendings and residents) wrote the 600 encounter notes about 507 patients. Most patients were identified in the medical record as female (n = 350 [69%]). Most patients were identified as Black/African American (n = 406 [80%]), and 76 (15%) were identified as White.

### Negative Language in Medical Records

We identified 5 categories of negative language (Table 1). These categories were not mutually exclusive.

**Table 1.** Negative Language Categories^a^

Categories	Definitions	Examples^b^
Questioning credibility	Implication of physician disbelief of patient reports of their own experience or behaviors	He insists the pain is behind his knee. He claims that nicotine patches don’t work for him. I listed several fictitious medication names and she reported she was taking them, and that she takes “whatever is written there”
Disapproval	Highlights poor reasoning, decision-making, or self-care, usually in a way that conveys the patient is unreasonable	Reports that if she were to fall, she would just “lay there” until someone found her He was adamant that he does not have prostate cancer because his “bowels are working fine.” Counseled that there is no evidence for this, but patient has strong beliefs. She is adamant that she cannot perform any kind of exercise due to pain and will not change her diet.
Stereotyping	Quoting African American Vernacular English	Chief complaint - “I stay tired” Reports that the bandage got “a li’l wet”
	Quoting incorrect grammar or unsophisticated terms	States that the lesion “busted open” Reports she was unable to fill prescription for the “sugar pill”
Difficult patient	Inclusion of details with questionable clinical significance that depict the patient as belligerent or otherwise suggests that the physician is annoyed	She persevered on the fact that “a lot of stuff is going on at home with my family” but that “you wouldn't understand.” I informed her that this is unlikely to be helped by antibiotics and talked about smoking cessation with her. She said she will ask her ‘sinus doctor’ for antibiotics.
Unilateral Decisions	Language that emphasizes physician authority over patient	She was told to discontinue… I have instructed him to…

^a^ Note categories are not mutually exclusive and often overlapping.

^b^ Examples in tables are from actual encounter notes in the study.

#### Questioning Patient Credibility

Several patterns of language suggested disbelief of patient reports, either by implying a lack of patient competency to remember and convey accurate information, or by questioning the patient’s sincerity. Common topics about which physicians conveyed doubt were the genuineness of patients’ symptoms or their adherence to treatment. Physicians sometimes used explicit doubt markers (eg, “supposedly,” “claims,” or “insists”). For example, one physician wrote, “apparently he was sitting at home on the floor feeling fine when suddenly he felt fatigued all over his body,” and another physician wrote that the patient “insists she gets sick from vaccines.”

In addition to explicit doubt markers, physicians sometimes quoted aspects of the patient’s history or belief system in a way that could be interpreted as questioning the legitimacy of the quoted text, a tactic known as a scare quote.^25^ For example, one physician wrote, “he claimed it was from ‘fluid in my knee,’” and another physician wrote, “She takes albuterol for ‘chronic bronchitis.’” In this latter example, the quotation marks simultaneously cast doubt on the diagnosis of chronic bronchitis and implicate the patient as a person with inaccurate beliefs about her condition.

#### Disapproval

Physicians used language suggesting disapproval of the patient by highlighting poor patient reasoning, decision-making, and behaviors. Emphasizing poor patient reasoning, one physician wrote, “she has stopped eating fruit in the last month because ‘it could have killed her.’” By using this quote, the physician highlights the patient’s health beliefs as unorthodox and simultaneously characterizes her as overreacting. In terms of decision-making, one physician wrote, “He is well aware of increased risk of seizure and is willing ‘to take the risk.’” The use of quotation marks here serves no clear purpose other than to highlight that the patient is exhibiting poor judgment.

Sometimes, physicians conveyed negative judgment about the patient’s self-care, often related to adherence or other health behaviors. Language that simply and neutrally reported that the patient was not adherent (eg, “he has not been taking his blood pressure medication”) would not be categorized as disapproval and sometimes could even be categorized as positive if accompanied by context that explained the behavior from the patient’s perspective. Examples where patient behaviors were characterized with qualifiers that suggested disapproval included: (1) “Unfortunately she had neglected to refill her blood pressure medication over the last week.” (2) “She is still not interested in physical therapy at this time as it is ‘too much walking’ but otherwise would like to have a prescription for tylenol 3 which she had taken in the past.”

Finally, physicians sometimes used language that implied tiresome repetition (eg, “I again explained…” or “despite repeated counseling”*)* on the part of the physician. For example, a physician stated, “difficult to fully assess without glucometer or BG log, despite that we talked extensively about our need for it in previous appointment.”

#### Racial or Social Class Stereotyping

Occasionally, there was explicit racial or social class stereotyping where physicians would quote either African American Vernacular English, incorrect grammar, or nonstandard oversimplified medical terms. For example, one physician quoted the patient referring to a surgical bandage as having gotten “a li’l wet.”

#### Difficult Patient

Physicians sometimes gave details that portrayed the patient as ignorant or temperamental or suggested that the physician was frustrated with the patient. They used condescending or emotional language, such as “the patient was adamant” or “this seems to pacify him.” Physicians also sometimes used quotes in a way that might make the patient seem argumentative or unreasonable: “She will not consider taking it because ‘my heart is fine, I don't want you all messing with my heart.’”

#### Unilateral Decision Making

Sometimes physicians used language that conveyed a paternalistic tone, using phrases like “I have instructed her” or “I impressed upon her the importance of.” This language upholds the image of a power dynamic where the physician presumes authority and portrays the patient as childish or ignorant.

### Positive Language in Medical Records

We identified 6 categories of positive language (Table 2). These categories were not mutually exclusive.

**Table 2.** Positive Language Categories^a^

Name	Definition	Example^b^
Compliment	Explicit adjectives to describe patient positively	Mr. [Patient] is charming, pleasant, and kind. Mrs. [Patient] is a delightful female.
Approval	Highlighting patient knowledge, character, reasoning skills and self-care patient behaviors	She has a physical/mental robustness that belies her age. She remembers both recent and distant events and is enjoyable to converse with on many subjects. She struggled with quitting over the spring and summer but as of this clinic visit has quit tobacco for 1 week!! I provided much deserved praise and encouraged her to continue her trajectory.
Self-disclosure	Physician self-disclosure of their own positive emotions related to patient	I am happy to continue coordinating her care. I am also encouraged by his new spirit to improve his health.
Minimizing blame	Reports reduced patient capacity or unhealthy behaviors with patient-centered reasons that convey understanding and minimize blame	She has not been checking her morning glucose for a month because she lost her blood glucose monitor. She has not been taking iron because it makes her constipated.
Personalize	Incorporation of details about the patient as an individual or particular person	She is a song writer and also sings. She has a strong faith in God and believes that he has blessed her and continues to keep her strong in light of her progressive disease. She enjoys walking with her fiancé and her dog named Scout.
Bilateral decision making	References to the incorporation of patient preferences into the treatment plan	He does not want to add a medication so I will increase the dose. She stated that even if it was positive, she would not want further testing. She will think about this and let me know next time if she wishes to proceed.

^a^ Note categories are not mutually exclusive and often overlapping.

^b^ Examples in the table are from actual encounter notes in the study.

#### Compliments

This category included explicit descriptions of patients using positive adjectives. For example, physicians described patients as being “charming,” “inspiring,” “pleasant,” and “kind.” These compliments were usually located at the beginning of the medical notes.

#### Approval

Physicians showed approval for positive patient behaviors, often for patients being active in their care or having achieved something difficult. For example, some notes contained phrases such as “I congratulated the patient on her hard work” or “he is very motivated and will likely be successful given the right resources.” Other physicians wrote, “she has quite good insight into her disease” and “patient is very knowledgeable about her medication.”

#### Self-disclosure

Physicians sometimes self-disclosed their positive emotions toward the patient. For example, physicians stated experiencing personal happiness, satisfaction, and encouragement. Examples included (1)“I am also encouraged by his new spirit to improve his health.” (2) “She is pleased with this development, as am I.” (3) “Patient expressed her gratitude for care the last few years and expressed her thanks. I … expressed my gratitude as well for being an inspiring patient.”

#### Minimizing Blame

Sometimes, patient notes seemed to have an overall positive tone even when the patient was not exhibiting adherence to treatment plans. In one instance, a physician described a patient as a “very pleasant male with multiple barriers to accessing healthcare.” In another case, a physician described that a patient “has limited short term memory that makes it difficult for her to carry out the interventions we recommend, even if they are limited in number.” Although this description questions the patient’s ability to convey an accurate history and engage in self-care, we did not classify this negatively as the physician gave the reasoning for why this patient may not be doing what they were advised, minimizing patient-blaming, and promoting understanding toward the patient. This contrasts with language previously described as conveying disapproval when the patient did not adhere to or agree with a recommended treatment plan.

#### Personalization

Patient notes sometimes included information that humanized the person by conveying details about the patient’s life from the patient’s perspective, such as the activities that the patient enjoys or the people who are important to them. For instance, a physician noted that “She is active, enjoys her independence, and likes to travel.”

#### Collaborative Decision Making

Finally, in contrast to unilateral decision-making, which emphasized physician authority and control and could come across as belittling the patient, physicians often used a tone in their assessments and plans that conveyed the plan was jointly decided or that the plan was directed by the patient. For example, physicians would write, “we discussed,” “he would rather,” or “she will consider.”

## Discussion

In this study, we described and classified linguistic features that may reveal negative and positive attitudes expressed in patients’ medical records. Physicians convey negative impressions in encounter notes by suggesting that the patient is not being truthful, expressing disapproval of the patient’s decisions and health-related behaviors, revealing racial or social class stereotypes of patients, displaying their own frustrations, and implying that the patient is unreasonable. Physicians also portray patients positively by using compliments, showing approval, self-disclosing their feelings of respect toward the patient, minimizing blame when patients are not adherent to treatments, and incorporating patient preferences into treatment plans. These sentiments portrayed in encounter notes are important to consider because they have the potential to influence the attitude and behavior of other clinicians reading those notes.^23^ The fact that nearly all medical centers in the United States have implemented electronic health records (EHRs) that make notes readily available to all health care clinicians within and across health systems underscores the scope and implications of these findings.

Patients who have difficult interactions with a clinician may perceive that they are not receiving high-quality, patient-centered care, and may be at risk of distrusting or disengaging from care. Stigmatizing language used to characterize those patients in their medical records potentially compounds this problem. Stigmatized patients may encounter clinicians in sequence, with each subsequent clinician treating them in accordance with the impressions expressed by the previous clinician. This reinforces and potentially confirms the patient’s belief that they are receiving inadequate care. Negative feelings may stay with the patient when moving between clinicians, eliciting their past negative emotions and experiences and transferring it to other clinicians, creating self-fulfilling prophecies and confirming stereotypes.^23,26^ The consequences of this self-fulfilling prophecy may be documented repeatedly in the medical record, perpetuating bias and inequitable care, and further disenfranchising the stigmatized patient.

Attendings and residents who staff ambulatory internal medicine clinics are often under time pressure and other stress,^27^ which can contribute to bias activation, emotional frustration and burnout, all of which might exacerbate any tendencies clinicians might have to vent negative attitudes toward patients in the medical record. Addressing the underlying stress and frustration that many clinicians have in their practices may be among the most important ways to reduce expressions of disrespect toward patients. However, we believe an enhanced awareness of clinicians’ word-use patterns, and of the potential consequences of those patterns, may motivate many well-intentioned clinicians to make improvements in their own documentation practices. Improving language use to reduce its negative impact on patient care can be considered an element of clinicians’ commitment to professionalism.^28^

The linguistic patterns we described could potentially be coded into natural language processing algorithms to allow large-scale identification and categorization of potentially stigmatizing language in medical records. Quantification of stigmatizing language would enable researchers to study the impact of such language on patient care, and would allow health systems to evaluate its prevalence and use the data to implement efforts to improve the quality and patient-centeredness of medical record documentation. This is particularly important as patients increasingly access and read notes in their own medical records.^29^

It is worth noting that our team found it challenging to come to consensus about how to categorize some of the linguistic patterns we observed. We found ourselves second-guessing whether it was fair to categorize a particular statement as conveying a positive or negative attitude, when we could not be certain how the clinician felt when writing it. The valence of many of the statements we coded were subtle. But in the end, we recognized that bias is not likely to be highly explicit; stigmatizing language can be as covert as it is damaging, in the same way that other microaggressions are subtle and hard to prove.^30,31,32^ To account for the inability to know in many cases what the physician-writer’s intent was, we focused our analytic lens on how a clinician-reader might perceive or interpret the language being used. This approach gave our findings greater credibility, because we as readers could gauge our reactions to the language without having to guess what the clinician intended. It also focused our analysis on our primary goal, to describe how physicians’ language might influence other clinicians caring for the same patient. To further triangulate our findings, we presented these results to multiple physician audiences who generally agreed that the language examples conveyed negative and positive tones and also agreed that it was often difficult to know for sure what the author intended.

Some of the statements we coded as conveying negative attitudes could be characterized as having some relevance for relating to and caring for the patient in the future. However, while it may be argued that commenting on a patient’s demeanor or personality can have value for those interacting with them in the future, negatively characterizing patients can unfairly penalize them for a bad day. Negative characterizations may emanate more from the clinician’s frustration or bias than from any inappropriate behavior on the patient’s part, compounding the injustice of clinicians, who hold testimonial power, using such language to describe people in their permanent records.

Our research team included practicing clinicians, and we saw some of the statements we were analyzing as normative in the medical profession. For instance, we are often taught as clinicians to use patients’ own words, in quotes, to describe their symptoms in their own voice. We recognized, however, that while quotes can sometimes be used for that purpose, they are also often used in what have become known as scare quotes, which are intended to convey negative sentiments about a person.^25^ It would be highly disingenuous for us as clinicians to use scare quotes to convey negative attitudes, and then hide behind the convention of using quotes as a manifestation of patient-centeredness. At the same time, although some of the positive and negative language we have described might perceived by clinicians as simply the way we were taught to speak, it is worth questioning whether linguistic patterns that have become normative should continue as such. Much of the language we have learned and use comes from an era when paternalism was the dominant paradigm in patient-physician relationships. The fact that this language is considered normal does not mean it is also not harmful or denigrating.

It is also worth noting some of the complexities of positive attitude expression in patient medical records. The presence of compliments and praise in some patients’ records may raise concern that the use of any emotional language—negative or positive—widens a potential disparity between those who are regarded with a great deal of respect and those who are not. That line of reasoning might suggest that we should eliminate all emotional language, including compliments and approval. Another potential concern is that compliments (eg, pleasant) of patients who are Black, Indigenous, and people of color may reflect underlying racism associated with having lower expectations of finding those characteristics.^33^ On the other hand, the positive themes of minimizing blame, personalization, and collaborative decision-making reflect patient-centered attitudes that support the ideal of respect for patients that we believe clinicians ought to strive for in all interactions and notes. It is the biased application of these principles and language that is problematic, not necessarily its use per se.

### Limitations

There are several limitations to our study. First, the data for this study were collected at a time when patients technically had access to their records, but most had not yet engaged with their own EHR system. Therefore, physicians writing notes during this timeframe likely had no expectation that their patients would read the notes. However, studies have suggested that clinicians generally do not consider patient access to records when writing their notes.^34^ Second, our data were collected from an ambulatory, internal medicine setting at an urban academic medical center, which may limit generalizability of these findings to other specialties or settings. Third, we did not have data on the personal characteristics of the physicians, such as age, gender, race/ethnicity, or training status (resident vs attending). These characteristics—and racial/ethnic or gender concordance between patient and clinicians—may be important factors associated with how language is used and further research should explore this topic. In addition, we could not gauge the consequences of this language on patients’ experiences of care, nor its impact on the quality of subsequent care. Whether patients are able to detect the emotional and attitudinal tone of their clinicians and its influences on subsequent care should be examined in future studies. Finally, our research team could not know the clinician-authors’ attitudes (or subsequent readers’ attitudes), thus the results (and discussion) include many unverifiable assumptions.

## Conclusions

This qualitative study found that physicians express both negative and positive attitudes toward patients when writing encounter notes in the medical record. Stigmatizing language in patients’ medical records may reflect bias by the physician. Just as we have developed a greater understanding about microaggressions and micro-inequities,^30,31,32^ this study’s findings suggest that we must raise consciousness about how we write and read medical records. Future research should examine the extent to which this type of language is used and to which it differs by patient (eg, race/ethnicity or gender) or clinician (eg, level of training) characteristics, or by patient-clinician concordance, and should also seek to understand the impact of this language on patient outcomes. Language has a powerful role in influencing subsequent clinician attitudes and behavior. Attention to this language could have a large influence on the promotion of respect and reduction of disparities for disadvantaged groups.
